# Capacity building permitting comprehensive monitoring of a severe case of Lassa hemorrhagic fever in Sierra Leone with a positive outcome: Case Report

**DOI:** 10.1186/1743-422X-8-314

**Published:** 2011-06-20

**Authors:** Jessica N Grove, Luis M Branco, Matt L Boisen, Ivana J Muncy, Lee A Henderson, John S Schieffellin, James E Robinson, James J Bangura, Mbalu Fonnie, Randal J Schoepp, Lisa E Hensley, Alhassan Seisay, Joseph N Fair, Robert F Garry

**Affiliations:** 1 Tulane University Department of Microbiology and Immunology, New Orleans, LA, USA; 2 Autoimmune Technologies, LLC, New Orleans, LA, USA; 3 Corgenix Medical Corporation, Broomfield, CO, USA; 4 Vybion, Inc., Ithaca, NY, USA; 5 Tulane University Department of Pediatrics, Section of Infectious Disease, New Orleans, LA, USA; 6 Ministry of Health and Sanitation Workplace Health, Sierra Leone; 7 The Global Viral Forecasting Initiative, San Francisco, CA; 8 Kenema Government Hospital Lassa Fever Ward, Kenema, Sierra Leone; 9 Applied Diagnostics Branch, Diagnostic Systems Division, U.S. Army Medical Research Institute of Infectious Diseases, Fort Detrick, MD, USA; 10 Viral Therapeutics Branch, Virology Division, U.S. Army Medical Research Institute of Infectious Diseases Diagnostic Systems Division, Fort Detrick, MD, USA

## Abstract

Lassa fever is a neglected tropical disease with a significant impact on the health care system of endemic West African nations. To date, case reports of Lassa fever have focused on laboratory characterisation of serological, biochemical and molecular aspects of the disease imported by infected individuals from Western Africa to the United States, Canada, Europe, Japan and Israel. Our report presents the first comprehensive real time diagnosis and characterization of a severe, hemorrhagic Lassa fever case in a Sierra Leonean individual admitted to the Kenema Government Hospital Lassa Fever Ward. Fever, malaise, unresponsiveness to anti-malarial and antibiotic drugs, followed by worsening symptoms and onset of haemorrhaging prompted medical officials to suspect Lassa fever. A recombinant Lassa virus protein based diagnostic was employed in diagnosing Lassa fever upon admission. This patient experienced a severe case of Lassa hemorrhagic fever with dysregulation of overall homeostasis, significant liver and renal system involvement, the interplay of pro- and anti-inflammatory cytokines during the course of hospitalization and an eventual successful outcome. These studies provide new insights into the pathophysiology and management of this viral illness and outline the improved infrastructure, research and real-time diagnostic capabilities within LASV endemic areas.

## Background

Lassa virus (LASV), a member of the *Arenaviridae *family, is the etiologic agent of Lassa fever, an acute and often fatal illness endemic to West Africa. There are an estimated 300,000 - 500,000 cases of Lassa fever each year [1-3] with a mortality rate of 15%-20% for hospitalized patients, which can become as high as 50% during epidemics [4,5]. Presently, there is no licensed vaccine or immunotherapy available for prevention or treatment of this disease. Although the antiviral drug ribavirin is somewhat beneficial, it must be administered at an early stage of infection to successfully alter disease outcome, thereby limiting its utility [6,7]. Furthermore, there is no commercially available Lassa fever diagnostic assay, which hampers early detection and rapid implementation of existing treatment regimens (e.g. ribavirin administration). The severity of the disease, its ability to be transmitted by aerosol droplets and the lack of a vaccine or therapeutic drug led to its classification as a National Institutes of Allergy and Infectious Diseases (NIAID) Category A pathogen and biosafety level-4 (BSL-4) agent. Several imported Lassa fever cases have been described since 1973 primarily from foreign nationals displaying signs of the disease upon returning to native countries or having been evacuated after falling ill abroad [8-34]. To date, and despite the often severe nature of Lassa fever in Western African nations, resources have not been available to perform comprehensive daily analysis of blood samples from suspected and confirmed patients in-country. Continuous infrastructure improvements at the Kenema Government Hospital (KGH) Lassa Fever Laboratory (LFL) by Tulane University, Department of Defense (DoD) and the United States Army Medical Research Institute of Infectious Diseases (USAMRIID) since 2005 have allowed for the implementation of sophisticated diagnostic and research capabilities at this location. Currently the KGH LFL diagnoses Lassa fever using ELISA and lateral flow immunoassay (LFI) platforms to detect viral antigen and virus-specific IgM and IgG levels in the serum of every suspected case presented to the LFW. Additionally, the laboratory can assess 14 serum analytes using a Piccolo^® ^blood chemistry analyzer coupled with comprehensive metabolic panel disks. Flow cytometry powered by a 4-color Accuri^® ^C6 cytometer is used to perform immunophenotyping and intracellular and bead-based secreted cytokine analysis. Together, these diagnostic assays and instruments enabled the analysis of metabolic and inflammatory functions in real time utilizing the sera of individuals discussed in this case report with concomitant appropriate medical intervention.

The main patient case discussed in this report was closely monitored for nine days during his hospitalization, during which time his condition stabilized; he began walking with supervision and was nearing the end of ribavirin treatment. These studies herald a new era in real time diagnosis and management of Lassa hemorrhagic fever in resource poor, endemic areas of Western Africa. They represent a novel platform toward more efficient and broader control of the effects of this disease in the population at large.

## Methods

Human Subjects: Suspected LF patients, close contacts, and healthy volunteers were eligible to participate in these studies as outlined in Tulane University's Institutional Review Board (IRB) protocol for this project, National Institutes of Health/National Institutes of Allergy and Infectious Diseases guidelines governing the use of human subject for research, and Department of Health and Human Services/National Institutes of Health/National Institute of Allergy and Infectious Diseases Challenge and Partnership Grant Numbers AI067188 and AI082119. This project was approved by the Tulane University IRB. Adult patients in this manuscript have given written informed consent for the publication of their case details. Written informed consent was obtained from the adult guardian of patient G-1180 for publication of this case report.

Normal and positive control sera: Serum from one Sierra Leonean and two Caucasian American volunteers were used in these studies as normal controls. A serum sample collected from a 20-year-old pregnant woman who succumbed to Lassa fever in the KGH Maternity Ward on August 29, 2010 was used as a positive control. A single serum sample was collected from this subject before expiration, and assigned the coded designation G-1177.

Detection of Lassa virus antigen and Lassa virus-specific antibodies: Serum levels of Lassa nucleoprotein (NP)-specific antigen were first determined using LFI modules currently under pre-clinical development by Corgenix Medical Corp., Broomfield, CO, U.S.A., and the Lassa fever consortium (see acknowledgements). The LFI modules utilize LASV NP specific murine monoclonal antibodies in a capture line and gold-conjugated detection reagent. Downstream of the capture line is an anti-murine polyclonal control line. Serum samples are added to a sample well followed by buffer solution to initiate lateral flow through the reagent pads and across the capture and control lines. The formation of Lassa NP antigen immune-complexes by the reagents produces a red signal due to gold conjugate deposition, allowing visual interpretation or measurement by reflectance. The red signal can be seen within 3-5 minutes with full signal development between 15-25 minutes. The positive Lassa fever diagnosis is then confirmed with a sensitive antigen-capture ELISA employing either a murine monoclonal or caprine polyclonal capture antibody, followed by a peroxidase-labeled caprine reagent and tetramethylbenzidine (TMB) substrate. A standard curve was generated with recombinant LASV NP in order to quantify serum levels of virus-associated NP.

IgM and IgG levels to recombinant LASV proteins (NP alone and NP - glycoprotein 1 (GP1) - glycoprotein complex (GPC) combination) ELISA: Individual LASV proteins and combinations optimized for detection of virus-specific IgM and IgG levels in serum were coated in stripwell plates, blocked, dried, and packaged with desiccating packs (Corgenix Medical Corp.). For analysis, sera were diluted 1:100 in a proprietary sample dilution buffer and incubated in wells for 30 minutes at room temperature, washed, and incubated with optimized HRP-labeled anti-human IgG or IgM conjugates for an additional 30 minutes. After washing, detection was performed with TMB substrate for 10 minutes at room temperature, stopped with sulphuric acid, and read at A450 in a BioTek ELISA plate reader.

IgM and IgG endpoint titer determination: Sera were analyzed in 3-fold serial dilutions, starting at 1:50, in optimized LASV NP, GP1, GP2, Z protein combination ELISA, as outlined above. Reciprocal titers were calculated using mean +3 standard deviation (S.D.) cutoffs established with similarly diluted normal serum controls.

Piccolo^®^: The kinetics of fourteen serum analytes were analyzed daily using a Piccolo^® ^blood chemistry analyzer (Abaxis, Inc., Union City, CA) with Comprehensive Metabolic Reagent Discs. Normal values were determined for a male in the age range of the patient using established clinical guidelines. Blood was collected in serum vacutainer tubes from patients and control donors and allowed to coagulate for 20 minutes at room temperature, followed by centrifugation in a tabletop centrifuge. The serum fraction was collected for analysis and aliquots were stored in cryovials at -20°C for future use.

Flow Cytometry: The kinetics of eleven serum cytokines were analyzed daily using an Accuri^® ^C6 benchtop cytometer (Accuri Cytometers Inc., Ann Harbor, MI) with an eBioscience FlowCytomix Human Th1/Th2 11-plex Kit (Bender MedSystems GmbH, Vienna, Austria). Serum aliquots collected and frozen throughout the monitoring timeline were analyzed concurrently at the end of the study.

## Case presentation

### Presentation

**Figure 1:**
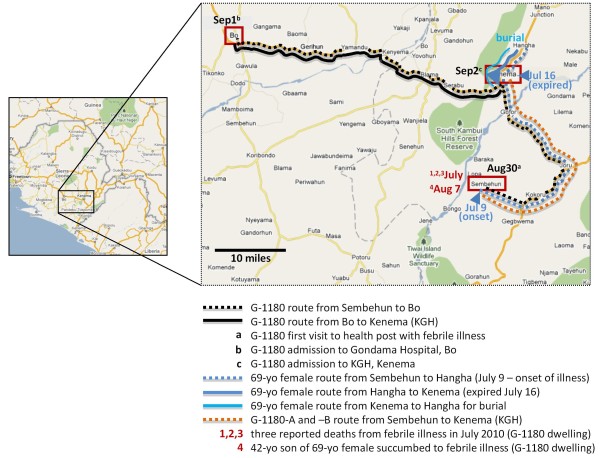
**Map of Sierra Leone and expanded view of Eastern Province, with relevant localities and routes travelled by patients in the current case report**. The Eastern Province of Sierra Leone is shown in the inset map with location of Sembehun, where all suspected Lassa fever cases in the current report originated, and the three localities where patients travelled to and from: Bo, Kenema, and Hangha. The inbound routes travelled by each patient are indicated in dotted lines, and outbound ones in solid lines. Three deaths from febrile illness in July were reported to the Outreach team during a local investigation in Sembehun following admission of G-1180 to KGH LFW. The travel history of a 69 year old female and her daughter from Sembehun to Hangha, and subsequently to Kenema, where she succumbed to a febrile illness without final diagnosis are highlighted. The 42 year old son of the 69 year old female reportedly succumbed to a similar febrile illness with hemorrhage on August 7 in Sembehun, without a final diagnosis. G-1180 was first transported to Ghondama clinic in Bo on September 1, after being referred by the local health post in Sembehun on August 30^th ^with fever and general malaise. The patient was subsequently transported to KGH LFW on September 2^nd ^after Lassa fever was suspected based on clinical presentation.

**Figure 2:**
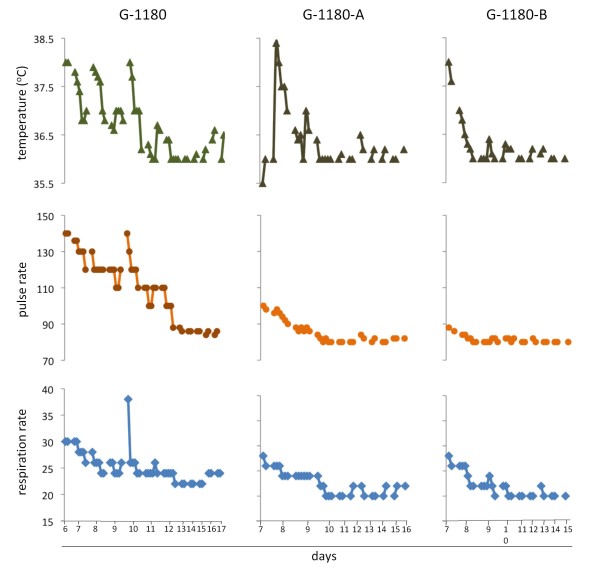
**Vital signs for G-1180 and contacts -A and -B during hospitalization at KGHLFW**. Core temperature (°C) [▲], pulse [●], and respiratory rates (per minute) [◆] were measured at regular intervals, usually every 4 hours at the onset, and every 12 hours at later times, throughout the hospitalization period for each patient. Data points were plotted individually and the normal ranges for each parameter are indicated by a line (temperature) or boxes (pulse and respiratory rates). A. Patient G-1180. B. G-1180 contacts -A and B.

Case 1: On September 1, 2010 the KGH LFW was contacted by the medical officer of Gondama Hospital in Bo district, Sierra Leone, concerning a suspected case of Lassa hemorrhagic fever. The patient was an eight-year-old male from Sembehun town, Malegohun chiefdom, Kenema district, Sierra Leone who had been first seen at the local health facility on August 30, 2010 (Figure 1). He presented to the health facility 2 days after the onset of symptoms with a history of persistent fever, headache, and profuse oral, nasal, and rectal bleeding. He was then referred to the Gondama Hospital where he could receive free medical care. After two days of treatment with anti-malarial and antibiotic drugs of unknown type and no improvement, the patient was referred to the KGH LFW as a suspected Lassa case. Upon arrival by ambulance at the KGH LFW on September 2, 2010, the patient reported having multiple symptoms including anorexia, malaise, headache, nausea, abdominal pain, loose black stools, hematemesis, dysuria, cough, sore throat and retrosternal pain. He presented with a body temperature of 38°C, a pulse rate of 140 beats/minute, and a respiration rate of 30 (Figure 2). On examination, he was in obvious pain and lethargic. Bleeding from the mouth and nose was noted along with conjunctival injection, facial edema and hepatomegaly. During the first 24 hours after his admission to KGH LFW, he had multiple episodes of grossly bloody stools as well as hematemesis, hemoptysis and hemeturia. This patient was assigned the coded designation G-1180 upon initial diagnosis by NP LFI at KGH LFW, which will be used henceforth.

### Clinical Chemistry

**Figure 3:**
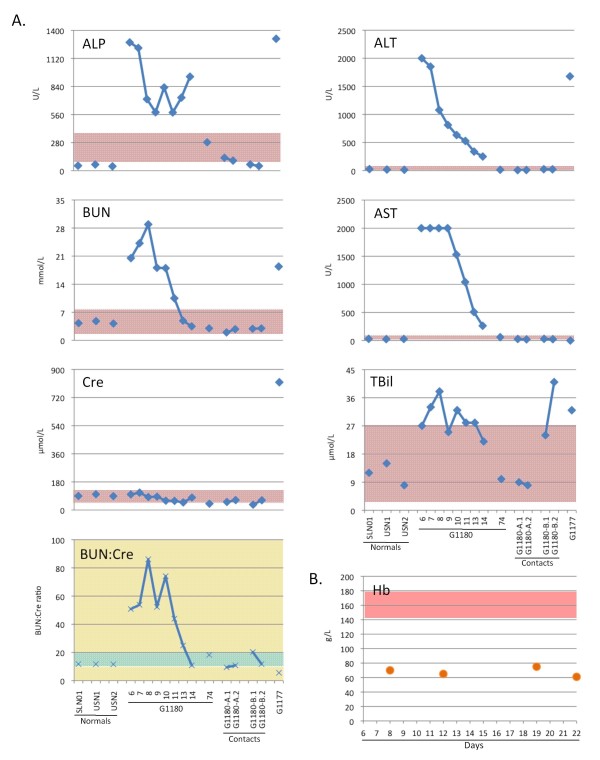
**A comprehensive metabolic panel was obtained daily by Piccolo analysis**. Fourteen metabolic indicators were measured in the serum of G-1180 daily after admission, through day 14 (with the exception of day 12), using a Piccolo comprehensive metabolic panel disk array (see additional file 1 figure S1 for Na^**+**^, Cl^**-**^, Ca^2+^, K^**+**^, TCO_**2**_, Alb and TP levels).A Two samples from G-1180 contacts -A and -B were also analyzed, along with Sierra Leonean and U.S. normal controls. Values were plotted alongside normal ranges for each metabolite, for reference (rose boxes). G-1180 presented with elevated TBil, and extremely high BUN:Cre ratios. Surprisingly, levels of Cre remained within normals levels throughout, in contrast to other report cases of Lassa hemorrhagic fever. The most dramatically elevated metabolic indicators were ALP, ALT, and AST, all indicative of severe liver implication in this case of Lassa fever. Patient G-1177, who succumbed to Lassa fever, presented with elevated TBil, but low BUN:Cre, mainly due to extremely high Cre levels. This patient also had highly elevated levels of ALP and ALT, but normal levels of AST. Metabolic indicators were assayed in normal Sierra Leonean and U.S. normals, along with two samples each from G-1180 contacts -A and -B. B Haemoglobin levels (Hb) were measured in G-1180 on days 8, 12, 19, and 22 post- onset of disease. Low levels of Hb prompted blood transfusions on days 9 and 14.

On presentation to KGH LFW, his sodium (Na^+^), chloride (Cl^-^), total carbon dioxide (TCO_2_), albumin (Alb) and total protein (TP) were all below the normal values for a male his age. The liver function panel revealed an aspartate aminotransferase (AST), alanine transaminase (ALT) and alkaline phosphatase (ALP) drastically above the normal range. Additionally, the blood urea nitrogen (BUN) and BUN: creatinine (Cre) levels were elevated. His potassium (K^+^), total bilirubin (TBil), corrected calcium (Ca^2+^) and Cre levels, on the other hand, all were within the upper normal range (Figure 3A, additional file 1 figure S1). His hemoglobin (Hb) levels were low throughout hospitalization (Figure 3B).

**Figure 4:**
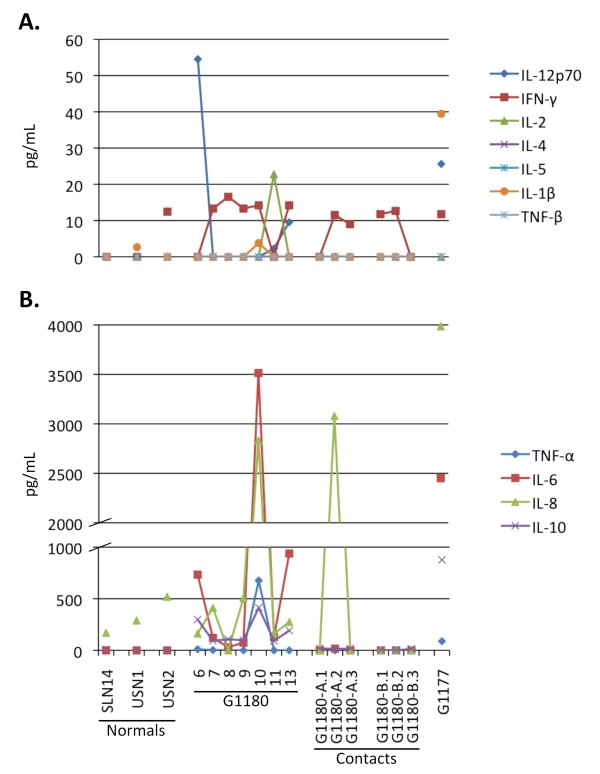
**Serum cytokine levels analyzed by multiplex Flow Cytometry**. Serum cytokine levels were analyzed with a BenderMed Systems Human 11-Plex Inflammatory Cytokine kit, and an Accuri C6 Flow Cytometer equipped with dual laser detection. Data was processed and quantitated with Flow Cytomic Pro software, and plotted on linear scales. G-1180 presented with elevated levels of IL-12p70, IL-6, and IL-10, all of which decreased by the following day. On day 10 post onset of disease a significant but transient spike in IL-1β, IL-6, IL-8, TNF-α, and IL-10 occurred, that rapidly decreased overnight, mostly to background levels. The only notable cytokine fluctuation in G-1180 contacts occurred in -A.2 with a dramatic spike in IL-8 that decreased to background levels within 2 days (-A.3). The single point analysis for G-1177 revealed high levels of IL-1β, IL12p70, IL-6, and IL-10.

At the time of admission to KGH LFW, a cytokine profile indicated that interleukin (IL) -12p70, IL-6 and IL-10 levels were elevated compared to the healthy controls (Figure 4).

### Diagnosis

**Figure 5:**
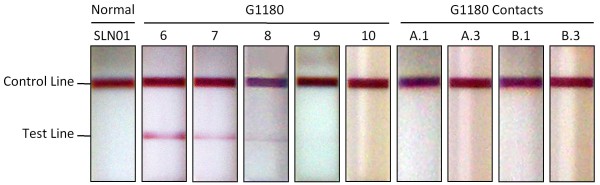
**Rapid diagnosis of acute Lassa fever virus infection by Lateral Flow Immunoassay (LFI) in patient G-1180 and contacts -A and -B**. LFI tests contain a murine monoclonal antibody capture and an heterologous gold-conjugated antibody for the detection of LASV nucleoprotein (NP) in the serum of suspected Lassa fever patients. Twenty-five μL of serum were applied to the sample well in an LFI module and chased with 3 drops (~ 100 μL) of optimally formulated buffer. After 10 minutes the results were recorded photographically. A representative normal serum sample analysis from a Sierra Leonean donor (S.L. N01) is shown for comparison. Only the control line developed with this serum sample. Conversely, sera from G-1180 generated a detectable precipitate in the test line, indicative of LASV NP antigen. The LFI platform detected NP antigen from days 6 - 8. Days 9 and 10 show no detectable antigen in this format. Sera from G-1180 contacts -A and -B did not produce a detectable signal on LFI, despite detection of antigen by a sensitive NP ELISA (figure 4A).

**Figure 6:**
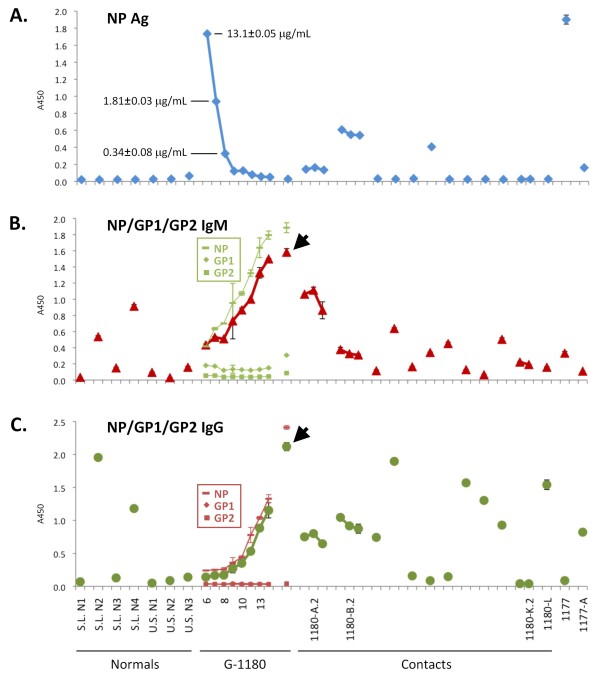
**ELISA detection of LASV NP antigen and virus-specific immunoglobulins M and G in normal donors, G-1180, G-1180 contacts, and in two additional patient and contact sera (G-1177 and G-1177-A)**. An antigen capture ELISA designed with affinity-purified goat polyclonal antibody reagents was used to detect LASV NP in patient sera (A). LASV NP antigen was not detected in normal sera from Sierra Leone and U.S. origin, or in most G-1180 contacts. The level of LASV NP antigen [◆, blue] in G-1180 dropped significantly during the first 3 days of ribavirin administration (quantitative levels indicated), to nearly undetectable levels by day 11. Conversely, antigen levels in G-1180-A and –B did not drop over the course of three days of ribavirin treatment. G-1180-F registered a significant level of antigen but did not seek treatment. G-1177 succumbed to acute Lassa fever, with very high levels of viral antigen detected before expiry. LASV-specific IgM (B) [▲, red] and IgG (C) [●, green] were detected in a recombinant ELISA plate format, coated with NP, GP1, and GP2, or with individually coated proteins. Most Sierra Leonean sera showed significant levels of IgM, IgG, or both, whereas U.S. normals did not. IgM and IgG levels in G-1180 rose throughout the course of the illness, and remained high on day 74 post onset of infection (black arrow). For G-1180 data are also plotted with IgM (▬ NP, ■ GP1, ◆ GP2, green) and IgG (▬ NP, ■ GP1, ◆ GP2, red) responses to individual LASV proteins. The IgM and IgG responses were directed primarily against NP, with a low IgG titer to GP1detected on day 74. Data are plotted as mean ±SD, N=2.

A blood specimen collected on the day of admission was positive for Lassa NP antigen (Ag) by LFI (Figure 5), and positive by quantitative NP antigen-capture ELISA, with a level of 13 μg/mL NP (Figure 6A). Additionally, IgM levels to recombinant Lassa proteins (NP alone and NP, GP1, GPC combination) were determined by ELISA, with low but detectable levels of immunoglobulin to NP and GPC (Figure 6B). IgG levels were not above background detection upon initial diagnosis (Figure 6C).

### Treatment and hospital course

Intravenous (IV) ribavirin was administered upon LFI Ag positive diagnosis: a loading dose of 30mg per kilogram followed by 15mg/kg every 6 hours for 4 days and 7.5mg/kg every 8 hours for an additional 6 days. Broad spectrum antibiotics (ceftriaxone, ciprofloxacin and metronidazole) and anti-malarials (artemether and quinine) were also started. A single dose of dexamethasone was given. Five percent and 50% glucose boluses were given as needed. Upon slight recovery, the patient requested energy drinks, which were provided (Lucozade, a sports-like drink similar to Gatorade in composition). Despite therapy, G-1180 continued to experience bleeding abnormalities for the next two weeks including hematochezia and hemoptysis. Ciprofloxacin was restarted on the eighth day post onset of illness to treat a catheter-related infection of his penis, which was resolved shortly thereafter. The patient required a blood transfusion on day 8 of illness due to severe anaemia (Hgb = 7.0 g/dL, Figure 3B). The donor was a Caucasian American female of the same blood type (A+). A second blood transfusion was administered on day 21 of illness (Hgb = 6.5 g/dL, Figure 3B) from a type O+ Caucasian American male donor. On this day the patient was alert, responsive, able to walk on his own and had stopped bleeding altogether.

Blood samples were collected daily, except for day 12 of illness. Day 14 post onset of illness was the last day that diagnostic, metabolic and inflammatory tests were conducted on patient G-1180. The LFI tests detected LASV NP in G-1180 through day 8 of illness (Figure 5) and NP Ag capture ELISA detected LASV NP through day 11 (Figure 6A; P < 0.05, N = 2). LASV NP antigen dropped rapidly over 3 days following the onset of ribavirin treatment (Figure 6A).

Despite the severity of the disease, most of G-1180's chemistry levels were near or within the normal range for a male his age by the end of our monitoring period. The originally low levels of Na^+^, Cl^- ^and TCO_2 _returned to normal levels; though, the Alb and TP remained consistently low. The liver function panel decreased significantly, although it did not return to within normal range. BUN and BUN:Cre levels, on the other hand, returned to normal levels. The analytes that were initially within the normal range remained stable throughout the monitoring period except for K^+^, which declined.

Cytokine profiles were performed on serum samples collected daily. G-1180's IL-12p70, IL-6 and IL-10 levels were elevated upon admission (day 6) but returned to normal by day 7. On day 10, a sudden increase in IL-6, IL-8, IL-10 and tumor necrosis factor (TNF) -alpha levels was noted. These levels all decreased to background values the following day. IL-2 was increased on day 11 only. IL-6 was again elevated on day 13.

Two blood samples have been collected from G-1180 since his discharge from the KGH LFW. A blood sample for follow-up testing was obtained on day 74 post onset of illness. At that time, IgM titers against GPC and NP had risen relative to day 14. Endpoint titers, determined by NP, GP1, GP2 and Z combination ELISA diagnostics, revealed a three-fold increase in IgM titer and an eighteen-fold increase in IgG titer from day 6 to 14 post onset of ilness, and a three-fold increase in both IgM and IgG titer from day 14 to 74 (additional file 2 table S1). Additionally, his complete metabolic panel had returned to the normal range except for a slightly elevated Na^+ ^and TP, and low Cl^- ^and Ca^2+^. The liver function panel had also normalized. A second follow-up sample obtained on day 108 post onset of illness revealed slightly elevated Na^+ ^and K^+^, and low Cl^- ^(data not shown). The Cre level was below normal and both ALP and AST were slightly elevated (data not shown). TP also remained elevated since the previous analysis.

### Chemistries of Healthy Volunteers and a Fatal Case of Lassa Fever

In order to establish the capabilities and reliability of the Piccolo^®^, complete chemistries were performed on blood drawn from healthy volunteers as well as a single sample from a patient who succumbed to Lassa Fever. This last sample (G-1177) was drawn less than 12 hours prior to expiry.

The chemistries of the three healthy volunteers were in the normal range as specified by the manufacturer (Abaxis, Inc.) except for a few values. One subject had a low serum Na^+^, two subjects had low Cl^-^, one had a low TCO_2 _one had an elevated TP (Figure 3, additional file 1 figure S1).

Subject G-1177 had extremely abnormal labs prior to expiring. Many of her analytes were above the normal range specified by Abaxis, Inc., including Na^+^, K^+^, Cl^-^, ALP, ALT, TBil, BUN and Cre (Figure 3, additional file 1 figure S1). Conversely, the TCO_2_, Alb, BUN:Cre and TP levels were low. Only the corrected Ca^2+ ^fell within the normal range. The AST level was zero. When compared to healthy volunteers, she presented with high levels of circulating IL-1beta, IL-12p70, IL-8, IL-6, and IL-10.

### Contact Tracing

On the night of September 2^nd ^patient G-1180 was admitted to the KGH LFW and as routine procedure in the KGH Lassa fever program, the outreach team was dispatched to the village of origin to further investigate the case. Three prior deaths of relatives of G-1180 living in the same dwelling were reported to the outreach team at that time. These deaths were attributed to an undetermined febrile illness and all occurred within the previous month. The deceased family members included a 69-year-old woman, a 42-year-old man and a third individual whose symptoms before death were not known. The histories of the 2 known individuals are as follows: On July 9^th ^the 69-year-old female reportedly experienced persistent high fever, vomiting, diarrhoea and severe headaches. A few days after the initial onset of symptoms the woman travelled to Kenema to seek medical treatment. The 69-year-old woman was admitted at the Arab Hospital, Hangha Road, Kenema for treatment but succumbed on July 16, 2010 without definitive diagnosis. The body was subsequently transported to Hangha and buried at the town cemetery (Figure 1). Two weeks after the death of the 69-year-old woman, her 42-year-old son living in the same dwelling as G-1180 fell ill with similar presentations, including profuse bleeding from all orifices, and succumbed on August 7, 2010 without visiting a health facility. The 42-year-old man was buried at Sembehun Town cemetery. The history of the third, expired relative is unknown.

The outreach team performed a close investigation of the dwelling, which revealed rodent faeces and holes in the walls of all rooms, poor food and water storage and congested home settings. At this time, eight close contacts of G-1180 were identified and blood samples were collected and transported to KGH LFL for LASV Ag and IgM analyses. None of the subjects had a recent travelling history outside the township.

Two of the contacts (G-1180-A, female, age 20, and G-1180-B, female, age 38) tested positive for LASV Ag and IgM, and were subsequently transported to the KGH LFW for ribavirin treatment. Despite detecting LASV Ag and IgM in both patient sera, neither presented with classical symptoms of the disease. G-1180-A and -B, provided only three serum samples each during their 10 day stay in the ward on three consecutive days starting with date of admission on September 4, 2010. Patient G-1180-A was admitted with an elevated TP that declined during ribavirin therapy. Her Alb and Ca^2+ ^remained low. The daily cytokine profile was unremarkable when compared to the healthy volunteers except for a one day increase in IL-8 observed on day two. Patient G-1180-B developed several abnormalities during the 3 day monitoring period. Her TP and TBil increased drastically; though, her cytokine profile was unremarkable.

The outreach team conducted sensitization meetings with the community, including a close door conference with village heads and health committee members. General education on Lassa fever and preventative measures were conveyed to the community through a film on the subject. The team also set rodent traps during their overnight stay, but *Mastomys *species, the known rodent reservoir of Lassa fever virus, were not trapped. The team was informed that shortly after the onset of G-1180's illness, rat poison was applied in the dwelling.

## Discussion

The NP is the most abundant polypeptide in Lassa virions, with approximately 60 molecules per particle. Thus, detection of NP antigen may be the most sensitive diagnostic for LASV, particularly in samples presenting with low antigenemia. Thus LFI and NP Ag detection ELISA were developed into rapid and confirmatory diagnostic platforms, respectively. Additionally, sensitive assays for the detection of LASV-specific IgM and IgG (Corgenix Medical Corp.), based on recombinant LASV protein ELISA, were used to qualitatively measure immunoglobulin levels (Figure 6B, C). Data from these sensitive assays suggest that G-1180 was naive to LASV exposure prior to this incident as he presented with very low NP-specific IgM levels on day 6, which progressively increased throughout the monitoring period. Conversely, GP1 and GP2-specific IgM titers were not statistically above background levels throughout the same period. On day 10, low IgG titers were detectable in G-1180 as determined by endpoint titers, which increased three-fold through day 14. Immunoglobulins were again measured on day 74 post onset of illness in G-1180, and surprisingly IgM titers against glycoprotein and NP had risen relative to day 14, rather than subsiding (additional file 2 table S1). An expected increase in IgG titers to LASV proteins was also observed. Immunoglobulin levels in G-1180 measured on day 108 post onset of illness revealed still elevated IgM titers as well as IgG titers against NP (data not shown). The IgM titer to NP dropped to approximately one third the qualitative level measured on day 74, whereas IgG rose slightly compared to the same time point. IgM and IgG levels against GP1 and GP2 were not significant on days 74 and 108. This phenomenon of persistently high IgM titers against LASV antigens for weeks, months or longer, in a significant proportion of convalescent patients is currently under investigation (Garry et al., unpublished data).

**Figure 7:**
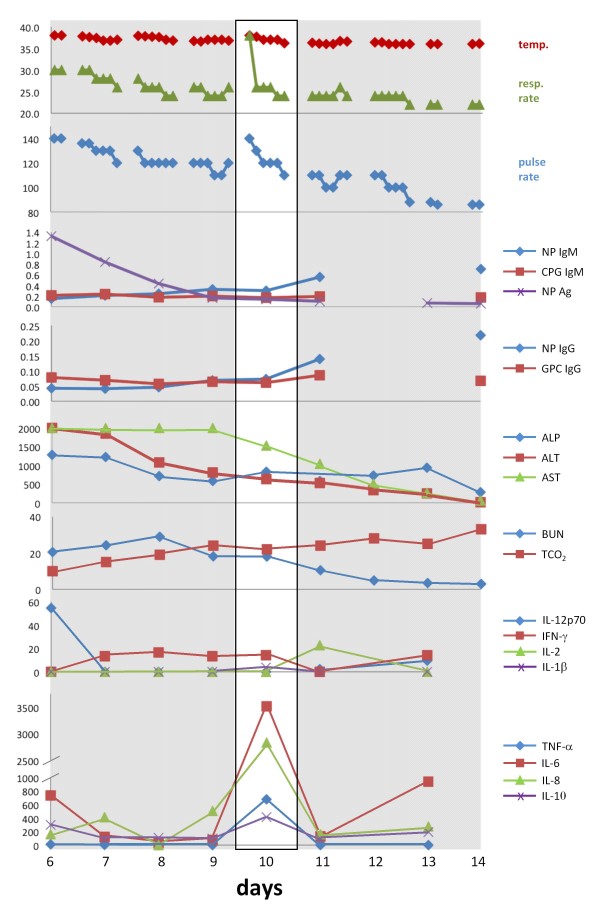
**Composite analysis of all parameters evaluated for G-1180 in the course of these studies**. Data for core temperature, respiration and pulse rates, LASV antigen, virus-specific IgM and IgG, metabolites, and cytokines were aligned for facile comparison of daily profiles. The single profile on day 10, which registered an increase in core body temperature to febrile levels, dramatic increase in respiratory and pulse rates early in the day, accompanied by elevated levels of pro- and anti-inflammatory cytokines is noteworthy, and is boxed in white.

The most remarkable aspect of the cytokine profile obtained at the time of admission was the significant level of IL-12p70 measured in serum (Figure 4). This pleitropic proinflammatory cytokine is produced by phagocytic cells, B cells, and other antigen-presenting cells that modulate adaptive immune responses, promotes Th1 cell development and is a potent inducer of IFN-γ and other cytokines in peripheral blood T and NK cells. IFN-γ further enhances production of additional IL-12 and other pro-inflammatory cytokines by phagocytic cells. IL-12 induced IFN-γ acts in a positive feedback loop providing an amplification mechanism in the inflammatory response [36]. Remarkably on day 7, IL-12p70 was not detected, but its decrease coincided with a rise in IFN-γ levels, which remained elevated through day 10, but were undetectable on day 11. Another increase in IL-12p70 levels was measured on days 11 and 12, the latter coinciding with a second spike in IFN-γ. Though IL-12p70 may have some impact on pathogenesis of Lassa fever, its role in arenaviral pathogenesis has not been described. Despite this profile early in the monitoring period other pro-inflammatory cytokines, such as IL-1β and TNF-α were undetectable, and IL-6 and IL-8 fluctuated in the lower end of the assay range. Elevated levels of IL-8 have been previously reported by Mahanty et al., 2001 to correlate with a positive outcome in acute Lassa fever infections [37], in addition to IFN-induced IP-10, which was not measured in these studies. A very notable albeit short increase in pro-inflammatory cytokines was measured on day 10, when IL-6 and IL-8 spiked to very high levels, in addition to a small spike in IL-1β and TNF-α. The increase of endogenous pyrogens IL-1β, TNF-α, IL-6, IL-8 on day 10 coincided with re-development of a mild fever, and significantly increased respiratory and pulse rates (Figure 7). These levels dropped to baseline or were undetectable 24 hours later. A single spike in IL-2, a cytokine important in the differentiation and survival of cytotoxic T cells, and a facilitator of immunoglobulins by B cells, was measured on day 11.

Concurrent with this observed increase in pro-inflammatory cytokines, a significant spike was measured in the IL-10 levels, an anti-inflammatory cytokine and potent inhibitor of Th1 cytokines and cellular responses. This spike coincided with the onset of a significant increase in the levels of LASV-specific IgM and IgG. Notably, IL-10 levels were elevated throughout the seven days of monitoring. Following the spike in pro- and anti-inflammatory cytokines, body temperature, respiration and pulse rates on day 10, the patient stabilized. His temperature did not return to febrile levels again during the course of these studies, and average respiration and pulse rates stabilized within normal levels. Thus, it appears that G-1180 experienced a dynamic interplay of pro- and anti-inflammatory cytokines and their physiological effects were ongoing throughout the extended analysis period of seven days. More importantly these results strengthen the hypothesis, as previously proposed by others [35, 37, others], that an imbalance between pro- and anti-inflammatory cytokines plays an important role in the development of Lassa hemorrhagic shock, with poor outcome. Notably, the marked absence of TNF-α, a potent inducer of endothelial damage via apoptosis [38] and thrombocytopenia [39] throughout the monitored course of illness in G-1180, with the exception of a spike on day10, suggests a somewhat regulated and effective immune response at play. This applies to the marked absence of IL-1β during the same period. It has been suggested that an early pro-inflammatory cytokine response followed by downregulation to baseline levels, which has been characterized in Ebola patients, may also be important in a regulated and balanced immune response and outcome in Lassa fever infections [40]. IL-4, a strong mediator of Th2 CD4+ T cells that stimulate B cells and enhance the humoral response, and IL-5, which functions as a B cell stimulator leading to increase in immunoglobulin secretion were not detected throughout the course of illness [36].

The two contacts of G-1180 that were admitted to the KGH LFW presented with lower, albeit significant levels of LASV NP antigen compared to G-1180 (Figure 6A). Surprisingly, and despite clear and re-confirmed presence of NP antigen in the serum of both G-1180-A and -B contacts by ELISA, levels did not decrease over a two day period following treatment with ribavirin. Neither contact presented with or developed classical symptoms of Lassa fever during their 10-day hospitalization period. Contact G-1180-A presented with very low but detectable IgM titer to NP, and background level titers to GP1 and GP2. G-1180-B presented with significant IgM titer to GP2, but undetectable titers to GP1 and NP. In both contacts IgG titers were detectable from the outset, but remained unchanged throughout the three days of testing according to endpoint titers. Thus, potential previous exposure to LASV and development of protective humoral and/or cellular immunity may have contributed to the observed Ag positive but asymptomatic status of these two patients

Despite the poor outcome for patient G-1177, a positive control used throughout these studies, she presented with moderate IgM to GP2, low IgM to GP1 and NP, high IgG to NP, and undetectable IgG to glycoproteins. Additionally, G-1177 presented with highly elevated levels of ALP and ALT, but with undetectable AST. Analysis of cytokines in the single G-1177 sample revealed similarities with those seen with G-1180. Notably, elevated levels of IFN-γ, IL-12p70, IL-6, IL-8, and IL-10 were detected. The most remarkable difference was a highly elevated level of IL-1β in G-1177, which was only detected in low levels in G-1180 on day 10. Similar to G-1180's profile, cytokines IL-2, IL-4, IL-5, TNF-α, and TGF-β were not detected in G-1177.

Together, this case outlines the severe and prolonged multi-organ dysregulation, pro- and anti-inflammatory cytokine up- and down-regulation, and difficulty in management of acute Lassa fever infections. This case also delineates the necessity for prompt treatment with IV ribavirn, fluid management and maintenance of electrolyte balance to counter hypovolemia, hemorrhagic shock and malnutrition during the early stage of infection. Given the health status of G-1180 on day 6 post onset of symptoms, this study points to the possibility of positive outcomes in Lassa fever patients with severely compromised metabolic and immunological presentations.

## Conclusions

Lassa fever has a high fatality rate if not diagnosed and treated promptly. Due to the low economic status of Lassa endemic areas, it often remains misdiagnosed and untreated. However, due to advances in diagnostics and laboratory research equipment at the KGH LFL, strides are being made towards better understanding the immunological impact of LASV. This case report exemplifies the utility of these methods in defining the host immune response to this pathogen. Our results combined with those of others [35,37-40] that extensively characterized metabolic and immunological parameters of Lassa fever, point to a strong divergence in metabolites, cytokines and immunoglobulin levels in positive versus fatal outcomes. Further studies on both the cellular immune response and cytokine profiles early in LASV infections will be critical in deepening our understanding of Lassa fever and in closing the gap that currently exists between the initial time of infection and diagnosis and treatment.

This case report represents a snapshot of severe Lassa fever and exemplifies the capabilities of KGH LFL in diagnosing and monitoring the immunologic response during and post infection. This report dispels misconceptions that these studies are impossible in field hospital settings and demonstrates the potential to deploy diagnostic capabilities that will drastically impact treatment of Lassa fever by allowing the rapid identification, isolation and treatment of Lassa fever cases. Lastly the data in this manuscript provides insights into the clinical course of Lassa fever. Not only will these data inform clinical case management but they will also be critical to validating animal models currently being utilized for testing new candidate therapeutics and vaccines.

## Competing interests

This work was performed as partial fulfilment of Ph.D. dissertation requirements for Jessica N Grove.

## Authors' contributions

JNG contributed to the experimental design, collected relevant data, performed data analysis, and drafted the manuscript. LMB contributed to the experimental design, collected relevant data, performed data analysis, and helped draft the manuscript. MLB, and IJM developed the LASV IgG, IgM, antigen capture ELISA, antigen capture LFI, and performed assay optimization. LAH manufactured recombinant proteins. JSS and JER critically reviewed the clinical data and helped draft the case report. JJB provided outreach support, performed community interviews and collected data in Sierra Leone critical for the draft of the case report. MF is the head nurse at the KGH LFW and was critical in providing patient status reports, and blood samples from afflicted patients, when medically advisable, throughout the study timeline. RJS, LEH and JNF contributed critical reagents and access to equipment at the KGH LFL and provided critical review of the manuscript. AS is Director PHC at MOHS Workplace Health, Sierra Leone. RFG is Principal Investigator of the Tulane Lassa fever program. He contributed to the experimental design and provided critical review of the manuscript. All authors have read and approved the final manuscript.

## Supplementary Material

Additional File 1 **Figure S1 - A comprehensive metabolic panel was obtained daily by Piccolo analysis**. Fourteen metabolic indicators were measured in the serum of G-1180 daily after admission, through day 14 (with the exception of day 12), using a Piccolo comprehensive metabolic panel disk array (see Figure 3A for ALP, ALT, AST, BUN, TBil, Cre). G-1180 presented with low serum Na^**+**^, Cl^**-**^, and Ca^2+^ions, with K^**+**^, acidotic (low TCO_**2**_) and low Alb and TP levels. Contrastingly, G-1177 presented with high Na^**+**^, Cl^**-**^, K^**+**^, low Ca^2+^, and normal TCO_**2**_. Metabolic indicators were assayed in normal Sierra Leonean and U.S. normals, along with two samples each from G-1180 contacts -A and -B. Click here for file

Additional File 2 **Table S1 - Determination of LASV Ag specific immunoglobulin M and G endpoint titers with NP, GP1, GP2 and Z combination ELISA**. IgM and IgG endpoint titers were determined for G-1180 and his contacts (A-L). G-1180 was admitted to KGH LFW with relatively low IgM and IgG titers that increased during hospitalization and upon convalescence. Contacts G-1180-A and -B presented to KGH LFW with significant IgM and IgG titers, suggesting previous exposure to LASV, and remained asymptomatic throughout the hospitalization timeline. Additionally, G-1180-C through -L displayed IgM, IgG, or dual immunoglobulin titers. Click here for file
