# Supplementary figures and images for: Capacity building permitting comprehensive monitoring of a severe case of Lassa hemorrhagic fever in Sierra Leone with a positive outcome: Case Report
Source: Virol J. 2011 Jun 20;8:314. doi: 10.1186/1743-422X-8-314 (PMC3283910; doi:10.1186/1743-422X-8-314)

## Slide 1
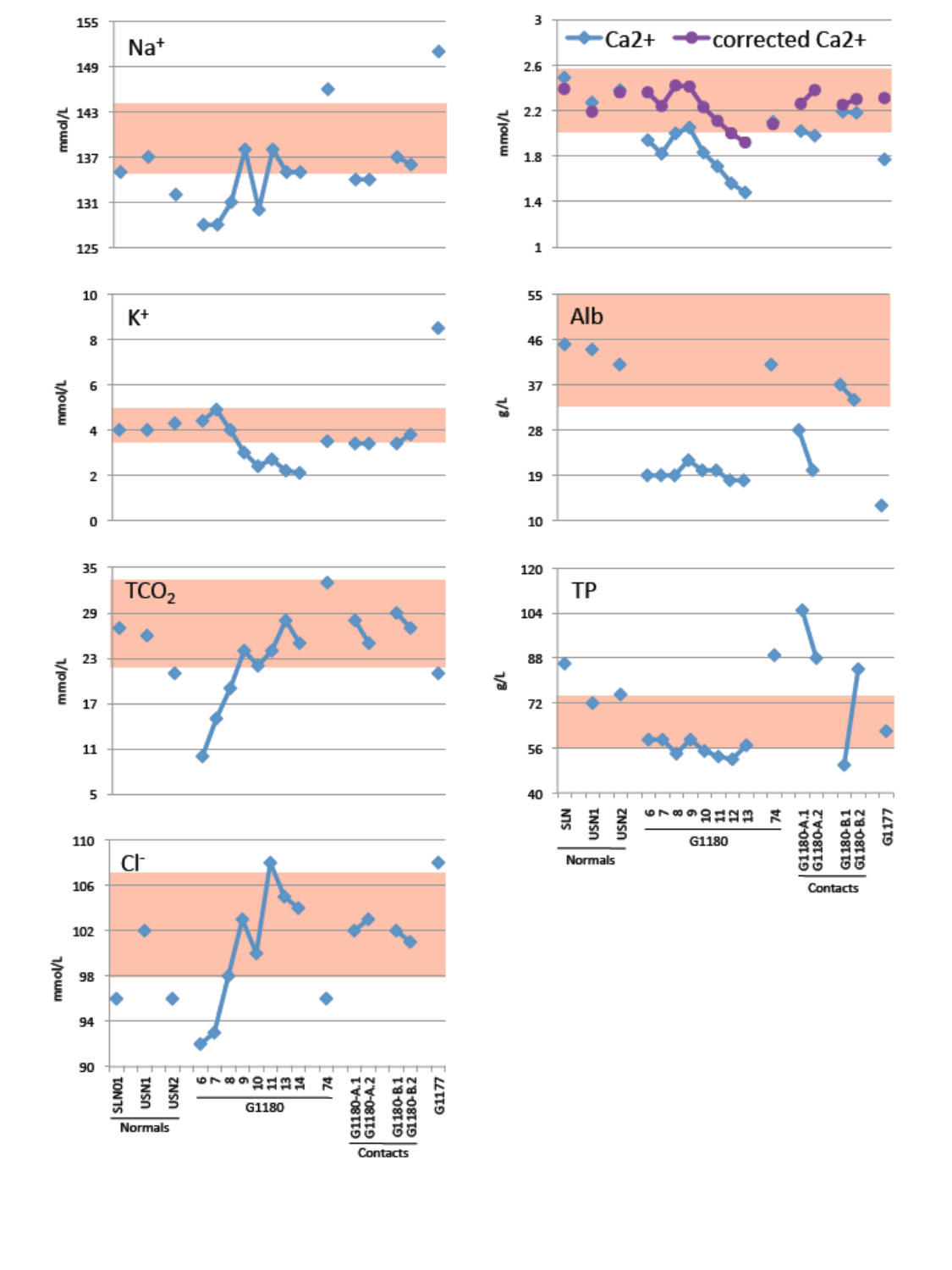

Supplement: Additional File 1 — Figure S1 - A comprehensive metabolic panel was obtained daily by Piccolo analysis. Fourteen metabolic indicators were measured in the serum of G-1180 daily after admission, through day 14 (with the exception of day 12), using a Piccolo comprehensive metabolic panel disk array (see Figure 3A for ALP, ALT, AST, BUN, TBil, Cre). G-1180 presented with low serum Na+, Cl-, and Ca2+ions, with K+, acidotic (low TCO2) and low Alb and TP levels. Contrastingly, G-1177 presented with high Na+, Cl-, K+, low Ca2+, and normal TCO2. Metabolic indicators were assayed in normal Sierra Leonean and U.S. normals, along with two samples each from G-1180 contacts -A and -B. [file 1743-422X-8-314-S1.PPT]

## Slide 1
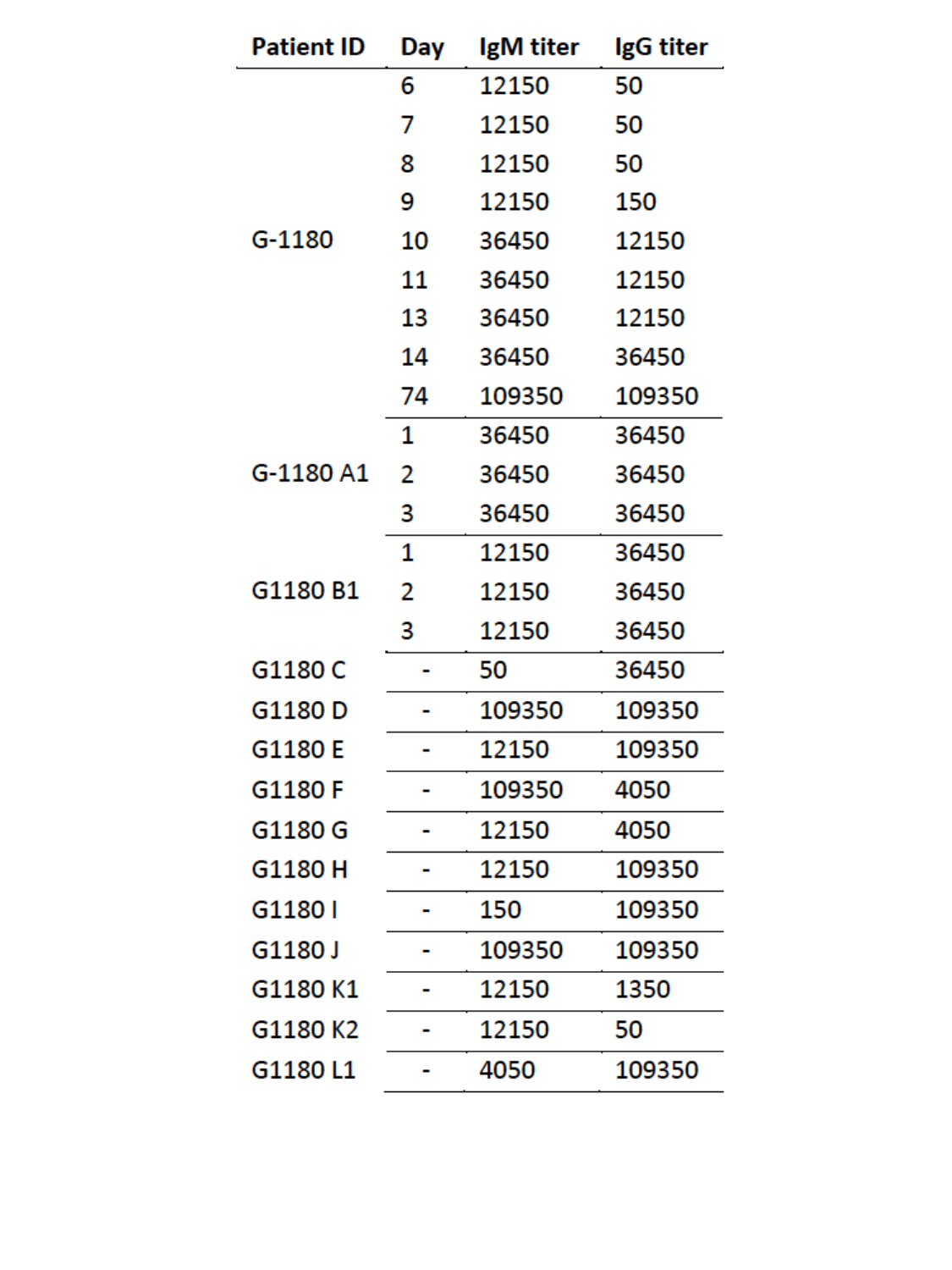

Supplement: Additional File 2 — Table S1 - Determination of LASV Ag specific immunoglobulin M and G endpoint titers with NP, GP1, GP2 and Z combination ELISA. IgM and IgG endpoint titers were determined for G-1180 and his contacts (A-L). G-1180 was admitted to KGH LFW with relatively low IgM and IgG titers that increased during hospitalization and upon convalescence. Contacts G-1180-A and -B presented to KGH LFW with significant IgM and IgG titers, suggesting previous exposure to LASV, and remained asymptomatic throughout the hospitalization timeline. Additionally, G-1180-C through -L displayed IgM, IgG, or dual immunoglobulin titers. [file 1743-422X-8-314-S2.PPT]
